# The physiological functions and therapeutic potential of exosomes during the development and treatment of polycystic ovary syndrome

**DOI:** 10.3389/fphys.2023.1279469

**Published:** 2023-11-06

**Authors:** Zhenghong Zhang, Congjian Shi, Zhengchao Wang

**Affiliations:** Provincial Key Laboratory for Developmental Biology and Neurosciences, College of Life Sciences, Fujian Normal University, Fuzhou, China

**Keywords:** exosomes, microRNA, oxidative stress, inflammatory response, insulin resistance, follicular development, polycystic ovary syndrome

## Abstract

Polycystic ovary syndrome is a very common disease of gynecological endocrine, accompanied by irregular menstruation, hyperandrogenism, metabolic abnormalities, reproductive disorders and other clinical symptoms, which seriously endangers women’s physical and mental health, but its etiology and pathogenesis are not completely clear. Recently, the contribution of exosomes to the diagnosis and treatment of various diseases in the biomedical field has attracted much attention, including PCOS. Exosomes are extracellular vesicles secreted by cells, containing various biologically active molecules such as cell-specific proteins, lipids, and nucleic acids. They are important signaling regulators *in vivo* and widely participate in various physiopathological processes. They are new targets for disease diagnosis and treatment. Considering the important role of non-coding RNAs during the development and treatment of PCOS, this article takes exosomal miRNAs as the breakthrough point for elucidating the physiological functions and therapeutic potential of exosomes during the development and treatment of PCOS through analyzing the effects of exosomal miRNAs on ovarian follicle development, hormone secretion, oxidative stress, inflammatory response and insulin resistance, thus providing new research directions and theoretical basis for PCOS pathogenesis, clinical diagnosis and prognosis improvement.

## 1 Introduction

PCOS is a common endocrine disease in the childbearing women. The main clinical manifestations are irregular menstruation, hyperandrogenism, metabolic abnormalities, ovulation dysfunction and the morphology of polycystic ovary. The global incidence rate is 4%∼21% (Teede et al., 2010; Lizneva et al., 2016; Azziz, 2018; Velez et al., 2021; Karjula et al., 2022), and has shown a significant upward trend in recent years. PCOS not only affects women’s reproductive and physiological health, but also can lead to the occurrence of metabolic and non-metabolic complications like hypertension, NAFLD, CVD, depression and anxiety (Anagnostis et al., 2018; Cooney and Dokras, 2018; Kakoly et al., 2019; Rodriguez-Paris et al., 2019; Sadeghi et al., 2022). Therefore, PCOS is not only a reproductive system disease, it has gone beyond the field of gynecology and obstetrics to involve all major systems of the whole body, seriously threatening women’s physical and mental health, affecting women’s quality of life, can cause the lifelong endocrine metabolic disorder, and has brought a heavy burden to society and families.

PCOS patients exhibit the high heterogeneity in clinical practice, making it difficult to be elucidated with a single factor (Teede et al., 2010; Escobar-Morreale, 2018; Sadeghi et al., 2022). IR is a major feature of PCOS, which can lead to compensatory hyperinsulinemia, and then cause hyperandrogenism by stimulating androgen secretion and inhibiting the production of SHBG (Wang et al., 2017a; Wang et al., 2017b; Jeanes and Reeves, 2017). A research has shown that adipose tissue dysfunction may be a key trigger for IR in PCOS (Villa and Pratley, 2011; Almalki, 2021; Li et al., 2022a). Abnormal follicular development and gonadotropin production, especially the excessive secretion of LH, can also cause the occurrence and development of PCOS (Goodarzi et al., 2011; Escobar-Morreale, 2018). The existing researches have shown that the mechanism of PCOS ovarian dysfunction mainly involves two aspects: One is the imbalance of steroid hormone production, including congenital thecal cell dysfunction and adrenal cortex androgen dysfunction, the other is the dysfunction of GCs and the disorder of follicular development, including excessive androgen as well as the morphology of polycystic ovary (Rosenfield and Ehrmann, 2016; Wang et al., 2017b; Xu and Wang, 2021; Wang et al., 2023). PCOS pathogenesis is complex with the abnormalities in HPO/HPA endocrine and metabolism (Barthelmess and Naz, 2014; Meier, 2018; Dinsdale and Crespi, 2021). At present, it is widely believed that the abnormal changes in PCOS are mainly caused by environmental and genetic factors (Urbanek, 2007; Merkin et al., 2016; Dinsdale and Crespi, 2021). The treatment needs to be tailored to different patients, including improving hyperandrogenicity symptoms, inducing ovulation, regulating menstruation, and preventing myocardial metabolic complications (Jayasena and Franks, 2014; Escobar-Morreale, 2018). Therefore, it is currently necessary to further analyze its etiology from a new perspective, such as extracellular miRNA, to provide new targets for its effective prevention and genetic intervention.

In recent years, exosomes and their contents have be proved for their contribution to the occurrence and development of various reproductive, endocrine and metabolic disorder, including PCOS (Chen and Liu, 2022). More and more attention has bee attracted for the role of exosomes in PCOS, which has shown exosomes and their ncRNA were involved in PCOS, especially the potential regulatory effects on the follicular development of PCOS (Machtinger et al., 2016; Kalluri and LeBleu, 2020). ncRNA includes lncRNA, miRNA, circRNA and siRNA, which can participate in various biological processes and play important regulatory functions (Beermann et al., 2016; Quinn and Chang, 2016). miRNAs are small single stranded ncRNAs with 19–25 nucleotides. They can regulate the gene expression after transcription for silencing of target genes. miRNAs can target hundreds of mRNAs and simultaneously affect the expression of associated genes on certain specific pathways (Lu and Rothenberg, 2018). miRNA sequences are highly conserved, which regulate various biological functions of different organisms, such as cell apoptosis, proliferation, differentiation, and development (Long et al., 2014). miRNAs play a key role in almost all cells, and their abnormal expressions are related to many diseases including AID, cancer and infertility (Zhang et al., 2020b; Li et al., 2020b; Kiani et al., 2019. During the biosynthesis process, miRNAs can be released into intercellular spaces, making them detectable in body fluids like blood, urine and semen (Kiani et al., 2019; Saliminejad et al., 2019; Smolarz et al., 2022). miRNAs not only exhibit significant stability in various body fluids, but also reflect the secretion and metabolic activities of oocytes and follicular walls (Cirillo et al., 2019). Therefore, miRNAs are closely involved in PCOS pathogenesis.

With the rapid biotechnology, the development potential of exosomes in clinical applications continues to expand, especially in the rapid progress of disease diagnosis and drug treatment. Clinical studies have shown a correlation between PCOS and exosomes, which have a profound impact on the occurrence and treatment of PCOS. However, PCOS pathogenesis is not yet clear. In order to elucidate the physiological function and therapeutic potential of exosomes during the occurrence and treatment of PCOS, this article takes exosomal miRNAs as the entry point, and then analyzes the effects of exosomal miRNAs on ovarian follicle development, hormone secretion, oxidative stress, inflammatory response, and insulin resistance. Finally, the new research directions and theoretical basis are provided for PCOS pathogenesis, clinical treatment, and prognosis improvement.

## 2 The origination of exosomes

Exosomes are an extracellular vesicle (EV) with a lipid bilayer membrane structure of uniform size and 30∼150 nm in diameter that is actively secreted by cells. They contain a variety of biologically active molecules such as cell-specific RNA, DNA or proteins, which transmit genetics to the receptor and affect their normal cell functions (Sun et al., 2019; Khalyfa et al., 2020). Exosomes are distributed in many bodily fluids, like uterine cavity fluid and follicular fluid, adjusting many physiological processes (Simon et al., 2018).

**FIGURE 1:**
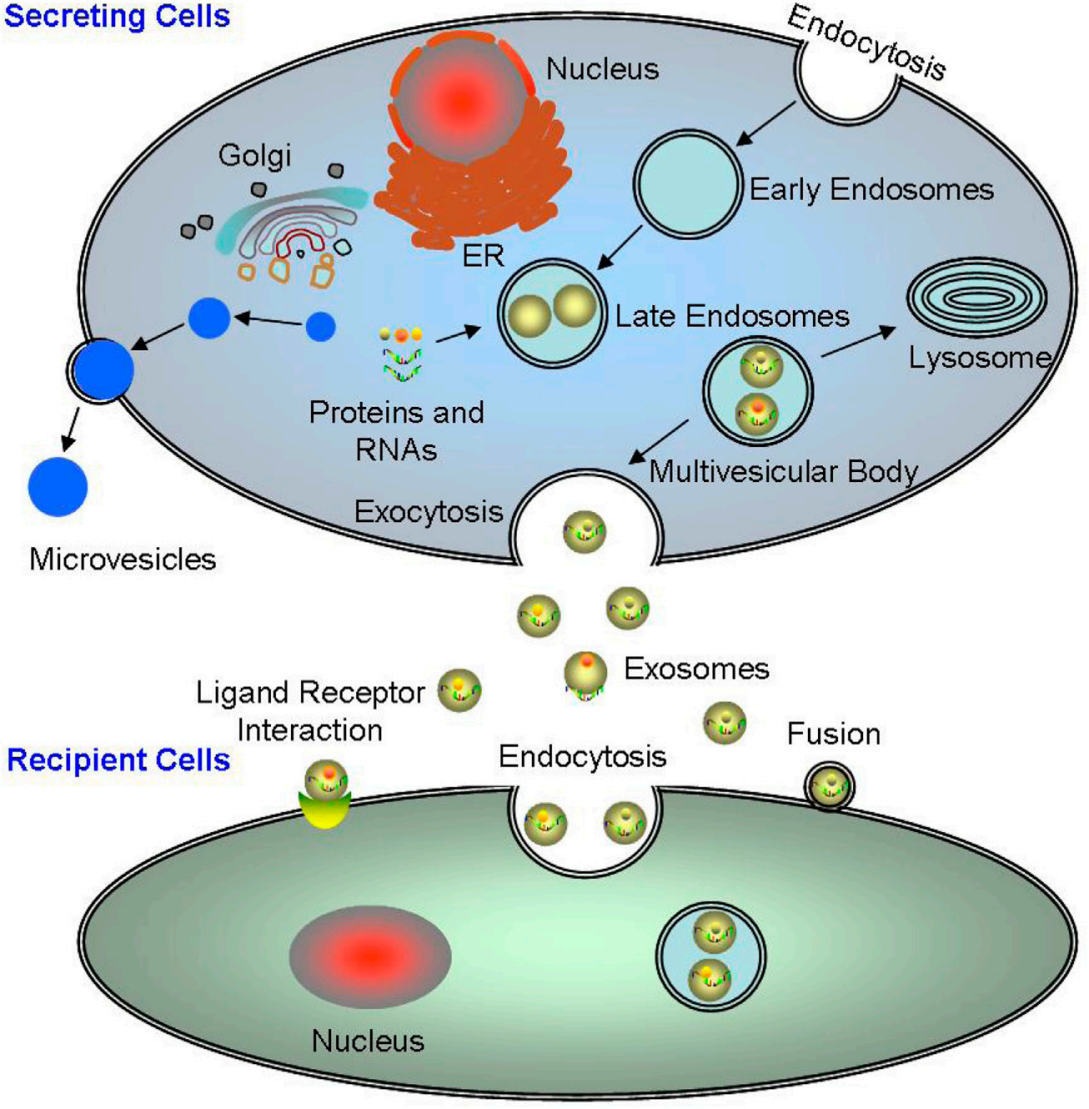
The origination and excretion of exosomes. The biogenesis of exosomes is a continuous process, from the early endosomes to the polyvesicular bodies, and finally secreted into the extracellular environment in the form of exosomes. Exosomes mainly interact with target cells main through three ways: directly binding to the target cells; completely internalized into the target cells; and binding to the target cells through surface molecules.

Exosomes are the main type of EVs, which are a new mechanism of cellular communication, where all cells can synthesize and secrete EVs including eukaryotic and prokaryotic cells. The biogenesis of exosomes is a continuous process, where the cell membrane sinks inward to form early endosomes containing various membrane surface proteins, exogenous antigens, and lipids; then the endosomes collapse again in the cells, wrapping proteins, nucleic acids, etc. in the cytoplasm to form vesicular bodies; lastly, the polyvesicular bodies fused with the cell membrane and exocytosis, and finally secreted into the extracellular environment in the form of exosomes (Figure 1) (Kalluri and LeBleu, 2020).

Exosomes can carry a variety of biomacromolecule substances, and these specific biological molecules contained in these exosomes determine their functions and are widely used as the biomarkers (Yang et al., 2019). In order to better understand the biological functions of exosomes under various physiological and pathological conditions, various isolation methods for exosomes have been established (Table 1) (Mashouri et al., 2019; Pegtel and Gould, 2019; Yang et al., 2019; Zhang et al., 2020c; Liang et al., 2021; Kimiz-Gebologlu and Oncel, 2022).

**TABLE 1:** Separation principle and characteristics of exosomes.

Separation method	Separation principle of exosomes	Advantages and disadvantages
Ultracentrifugation method	It is currently a commonly used method for purifying extracellular vesicles. Firstly, the follicular fluid was centrifuged at 300 *g* for 10 min at 4°C, followed by 2,000 g for 20 min. After discarding the precipitate, it was centrifuged at 10,000 g for 30 min before discarding the precipitate again. Finally, after discarding the supernatant at 10,000 g, the precipitate obtained was called exosomes	The advantage of this method is that it has a large amount of extraction, while the disadvantage is that the extraction purity is insufficient, the recovery rate is unstable, and repeated centrifugation may damage the vesicles. Under electron microscopy, the extracellular vesicles aggregate into blocks
Filtration centrifugation method	The filtration centrifugation method uses the separation principle of the pore size of the biofilm to centrifugate the sample to obtain exosomes, that is, use the ultrafiltration membrane to leave large molecules while small molecules can be filtered out through the ultrafiltration membrane	The advantage of this method is time-saving and efficient, and it is not easy to damage the exosomes; The disadvantage is that the purity obtained is relatively insufficient, and it is prone to residual adhesion on the ultrafiltration membrane, reducing the lifespan of the membrane. The production of exosomes in this method is relatively low, which is insufficient to support the conduct of experiments that require a large number of exosomes
Immunomagnetic Bead Method	On the basis of centrifugation, the sample to be tested is mixed with antibody coated immune magnetic beads, incubated at room temperature, and then slowly added to the separation column. The separation column is then removed from the magnetic separator and rinsed with phosphate buffer solution	This method is simple to operate and has high separation purity, which can ensure the integrity of exosome morphology. However, non-neutral pH values can affect the biological activity of exosomes and are not suitable for the separation of large volume samples. Therefore, it is not conducive to the subsequent functional experiments and the promotion of this method
ExoQuickPrecision Method	Using the ExoQuick reagent kit, centrifuge the follicular fluid at 3,000 xg for 15 min to obtain the supernatant. Add a precipitant (to reduce the hydration of the follicles and change their solubility) and centrifuge again to discard the supernatant. Complete exosomes are obtained and impurities in the extracted sample can be effectively removed	This method mainly relies on polymer co precipitation to obtain richer extracellular vesicles. However, its biological activity is easily affected by reagent concentration and pH, and the reagent kit is expensive, making it unsuitable for large-scale exosome extraction
Spiral Ultrafiltration Method	Spiral ultrafiltration method uses a spiral ultrafiltration instrument to extract extracellular vesicles. Firstly, the collected follicular supernatant was placed in two 15 mL centrifuge tubes, and centrifuged at 300 *g* and 2000 g at 4°C to obtain the supernatant. After filtration, the supernatant was repeatedly ultrafiltration by a spiral ultrafiltration device three times, and rinsed repeatedly with phosphate buffer solution. Finally, it was placed in an Eppendorf tube and stored in a −80°C refrigerator for future use	This method can obtain extracellular vesicles quickly and efficiently, and is commonly used
Size Exclusion Chromatography	Molecular exclusion chromatography separates the vesicles from other molecular phases through gel filtration. The gel is mainly composed of spherical particles, which are distributed with pores at specific positions. When the sample enters the gel, the small molecules can diffuse directly into the pores, while the large molecules are eluted directly, so the large molecules are easier to separate than the small molecules	This method utilizes complex biological characteristics to achieve the separation of extracellular vesicles, which has only recently been applied to the separation of vesicles. In the actual operation process, factors such as equipment type, pore size, and flow rate should also be taken into account to obtain exosomes with high purity and good functionality
Quantitative Detection Method	The collection of extracellular vesicles is first separated and enriched through methods such as centrifugation and immunomagnetic beads. However, due to insufficient purity and lack of recognized separation methods, quantitative research on extracellular vesicles is greatly limited. This article collects multiple methods for extracellular vesicle extraction for comprehensive analysis. Nanoparticle Tracking Analysis is a quantitative analysis method that utilizes the physical properties of extracellular vesicles to detect the distribution and concentration of nanoparticles using light scattering. It can simultaneously analyze samples of different sizes and distributions to minimize contamination in the processed samples	Electrochemical sensors are a method of quantitatively analyzing extracellular vesicles using their electrochemical properties. This method has the advantages of good characteristics, high sensitivity, and easy acquisition. Surface plasmon resonance technology can perform real-time quantitative detection of unlabeled extracellular vesicles, with high sensitivity and the need for fluorescence labeling, making it convenient and fast

## 3 Physiological functions of exosomes

**FIGURE 2:**
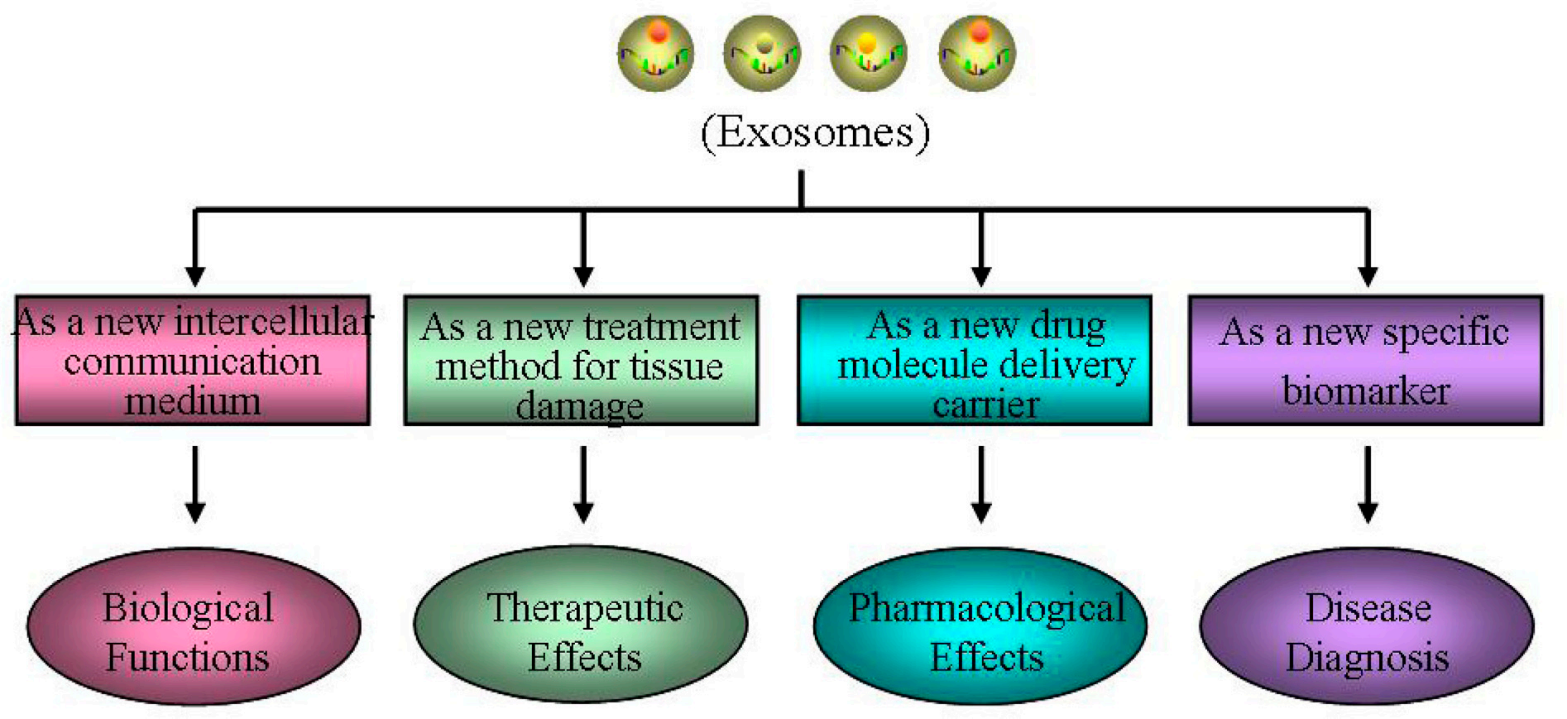
Different physiological functions of exosomes. Exosomes may have different physiological functions in various tissues under different physiological states, such as a new intercellular communication medium, a new treatment method for tissue damage, a new drug molecule delivery carrier and a new specific biomarker.

The RNA, protein, and lipid contents of exosomes from different cell sources are various, and their physiological functions are also different (Sun et al., 2019; Zhang et al., 2020c). Exosomes can directly activate target cells through plasma membrane receptors, or act as carriers to transport proteins, lipids, non-coding RNA, and even viruses into target cells, serving as signaling factors to alter the biological activity and function of target cells (Simon et al., 2018; Pegtel and Gould, 2019). Therefore, exosomes have different physiological functions in various tissues under different physiological states (Figure 2).

Exosomes, as a new intercellular communication medium, play an important biological role. The exosomes have good stability, and their lipid bilayer membrane structure can protect the endosomes from degradation or modification. Therefore, the cell specific biomacromolecules they carried can be transmitted to other cells as signaling molecule to change the biological function of receptor cells, which is an important way of cell-cell information transmission. Exosomes can also participate in maintaining various physiological processes in organisms, including cell quality control, inter-cellular communication, cell waste clearance, immune regulation, tissue repair, stem cell division/differentiation, neovascularization, and coagulation (Simon et al., 2018; Pegtel and Gould, 2019; Kalluri and LeBleu, 2020). Furthermore, exosomes can mediate signal transmission and molecular transfer, contributing to a series of normal physiological processes, including tissue homeostasis, development, aging, metabolic regulation, molecular transfer during pregnancy, and breastfeeding (Pegtel and Gould, 2019). In addition, exosomes may have the potential to cross tissue barriers (such as blood–brain barrier) through endocytosis (Yuan et al., 2017).

Exosomes, as a new treatment method for tissue damage, have important clinical therapeutic effects. The specific biological molecules contained in exosomes determine the function of exosomes. In many fields, such as cancer, metabolic diseases, cardio cerebral vascular disease, and neuro degenerative disease, it mainly plays the role in regulating inter-cellular information transmission, immune response, and repair regulation, especially in mediating the progress and metastasis of cancer, and the early diagnosis and treatment (Pegtel and Gould, 2019; Kalluri and LeBleu, 2020). Giovannelli et al. (2021) reported that prostate cancer cells directly act on stroma or prostate cancer epithelial cells by releasing exosomes for cell communication. Wortzel et al. (2019) found that exosomes existing between primary tumor cells and distant organ microenvironment may promote the formation of local tumor micro-environment through mediating inter-cellular communication. Li et al. (2022b) found miR-155-5p, an exosome derived from tumor cells, can inhibit the progression of ovarian cancer and macrophage infiltration in immune intact mice, activate CD8^+^ T-cell function, and generate immune responses. In addition, the function of exosomes is highly heterogeneous due to the influence of cell sources and contents. Among them, exosomes derived from MSCs many be a novel treat method for tissue damage, affecting angiogenesis, tissue repairment, immune regulation, and resistance to inflammation.

Exosomes, as a new drug molecule delivery carrier, have important pharmacological effects. Based on its unique bio-compatibility, good stability and high affinity to cells, exosomes are regarded as the best carriers for chemical drugs, RNA, proteins and other drug molecules, playing a dominant role in preventing the decomposition of drug molecules carried, targeting receptor cells, and improving drug utilization (Peng et al., 2020). Lou et al. (2020) found AMSC exosomes modified by miR-199a-3p can improve the sensitivity of hepato-cellular carcinoma cells to chemo-therapeutic drugs through mTOR pathway, which to some extent solves the problem of easy degradation of miR-199a-3p molecules when directly administered *in vivo*. Kamerkar et al. (2017) have confirmed that the exosomes after genetic engineering transformation, which carry the miRNA-specific targeted oncogene KrasG12D, can promote the targeted therapy of the oncogene KRAS in pancreatic cancer, and show good efficacy in a variety of pancreatic cancer mouse models. Exosomes as drug delivery carriers have unique advantages, such as low immunogenicity, high transport efficiency, good stability, strong targeting, and ability to cross the blood-brain barrier (Yuan et al., 2017). At present, it has been reported that some small molecule chemicals and gene drugs have been successfully loaded into the exosomes, and have shown great potential in the treatment of nervous system disease and tumors (Haney et al., 2015).

Exosomes, as a new specific biomarker, play an important role in disease diagnosis. Exosomes can be stably transmitted in various bodily fluids and have become indicators of homeostasis in organisms. Their content reflects the fact that the originating cells and pathophysiological states highlight their effectiveness as biomarkers (Pegtel and Gould, 2019). Researches have demonstrated the differences of exosome contents between patients and healthy people, suggesting that they can be used as potential diagnostic indicators of diseases (Zhang et al., 2019c). At present, exosomes have already been proved to function in various human diseases, and many researches have shown that exosomes can be used as potential new diagnostic markers for many diseases, such as tumors (Sun et al., 2018b), degenerative disease (Quek and Hill, 2017), cardiovascular diseases (Jansen et al., 2017), and metabolic disorder (Akbar et al., 2019).

Together, it can be seen that exosomes are mediated by various stimuli, such as ultrasound (Zeng et al., 2019), ionizing radiation (Beer et al., 2015), DNA damage (Lehmann et al., 2008), enzyme effects (Zhang et al., 2018), and inflammatory stimuli (Almohammai et al., 2021). Under different pathological conditions, the release of exosomes are different, for example, OS can promote exosome release during ERS (Kanemoto et al., 2016). Exosomes mainly interact with target cells through the following three ways: 1) Exosomes directly bind to the target cells; 2) The exosomes are completely internalized by the target cells; 3) Exosomes bind to the target cells through their surface characteristic protein molecules (Figure 1). Exosomes participate in inter-cellular signal transduction, cell metabolism and proliferation (Zhang et al., 2019c; Wortzel et al., 2019). The nucleic acid carried by exosomes facilitates the angiogenesis and the rapid proliferation in the tumors (Sun et al., 2018b; Mashouri et al., 2019). Therefore, interfering the extra-cellular release of exosomes and disrupting the inter-cellular communication mediated by exosomes may be potential and powerful therapeutic strategies.

## 4 Role of exosomes in the pathogenesis of PCOS

During recent years, their contribution of exosomes to human reproductive health and diseases has received widespread attention (Hu et al., 2017; Pegtel and Gould, 2019; Sun et al., 2019; Zhang et al., 2021a). Exosomes can play important roles as key regulatory factors in different reproductive processes, such as follicular development and embryo implantation (Machtinger et al., 2016; Zhang et al., 2021a). As a disease with abnormal follicular development, studies have found the relationship of exosomes and PCOS (Hu et al., 2017; Simon et al., 2018). Koiou et al. (2013) found the positive relationship of ovarian follicles and plasma exosomes in a number-increased manner in PCOS patients. Jiang et al. (2021) found five miRNAs involved in estrous cycle, sinus follicle number, and hormone levels. Zhang et al. (2021a) reported significant differential expressions of four miRNA in PCOS serum exosomes, and identified these miRNAs as potential biomarkers. Recent researches have also found that multiple ncRNAs are abnormally expressed in PCOS ovaries (Sorensen et al., 2014; Li et al., 2015; Bahmyari et al., 2021; Zhang et al., 2022). These ncRNAs can mediate related-gene expression, affect hormone metabolism, and biological functions of GCs, thereby participating in the following regulation of the pathogenesis of PCOS (Fitzgerald et al., 2016; Bahmyari et al., 2021; Zhang et al., 2022).

**FIGURE 3:**
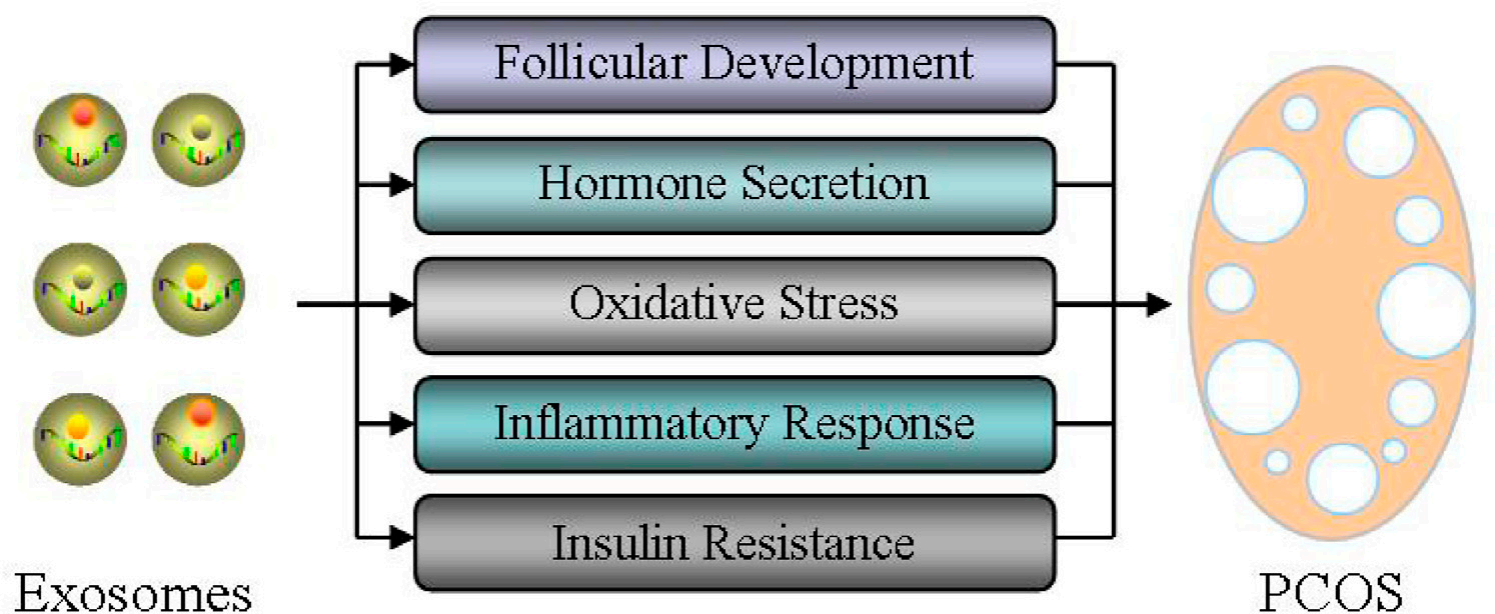
Role of exosomes in the pathogenesis of PCOS. The effects of exosomal miRNAs on ovarian follicular development, hormone secretion, oxidative stress, inflammatory response, and insulin resistance were analyzed in order to elucidate the physiological function and therapeutic potential of exosomes during the occurrence and treatment of PCOS.

Therefore, we here take exosomal miRNAs as a breakthrough point, and analyze their effects of exosomal miRNAs on ovarian follicular development, hormone secretion, oxidative stress, inflammatory response, and insulin resistance in order to elucidate the physiological function and therapeutic potential of exosomes during the occurrence and treatment of PCOS (Figure 3).

### 4.1 Effect of exosomes on follicular development

Abnormal follicular development induces anovulation, which is a fundamental feature of PCOS. During ovulation, matured oocyte is released after the follicle has been developed and ruptured, which is supported by the micro-environment of follicular fluid (Wu et al., 2013; Wu et al., 2015; Tang et al., 2017; Wu et al., 2016; Zhang et al., 2020a; Tang et al., 2021). Hu et al. (2020) showed the difference of exosome expressions in follicular fluid of PCOS patients, compared with those in the control group. These exosomes affect follicular development by regulating ovarian GCs and cumulus cells (Yuan et al., 2021; Zhao et al., 2022a).Yuan et al. (2021) found that miR-424-5p expressed inhibits the proliferation of ovarian GCs and induces the aging of ovarian GCs by blocking CDCA4-mediated Rb/E2F1 signaling pathway in PCOS. Zhao et al. (2022a) reported that in PCOS, exosomal miR-143-3p induces the apoptosis of ovarian GCs through blocking BMPRA-mediated Smad1/5/8 signaling. Xie et al. (2021) reported melatonin treatment regulates the autophagy of ovarian cells via PI-3K/Akt signaling, thereby improving ovarian dysfunction of PCOS rats. Additional studies suggested that miR-320a diminishes PI-3K-mediated insulin sensitivity because of their target p85 (Ling et al., 2009; Cirillo et al., 2019; Luo et al., 2021). Wang *et al.* reported the expression levels of miR-27a-3p, miR-23a, and miR-483-5p in serum exosomes of PCOS were upregulated, which may participate in regulating the growth of GCs and the synthesis of intrafollicular hormones in PCOS patients (Wang et al., 2019a; Wang et al., 2020). Therefore, exosomal miRNAs in follicular fluid are critical during the follicular development by influencing the biological functions of PCOS ovaries.

Abnormal function of GCs is an important cause of irregular development in PCOS follicles (Hussein, 2005). Sen et al. (2014) found that androgen can inhibit follicular atresia through up-regulating miR-125b expression in GCs, promoting the growth of preantral follicles, and reducing the expression of apoptotic proteins mediated by MAPK 1/3 and other signaling pathways. Jiang et al. (2015) reported that miR-93 is upregulated in PCOS GCs, and then regulates GC proliferation by targeting CDKN1A. Ding et al. (2020) found through miRNA microarray analysis that miR-9119 expression is upregulated, which can regulate the activity of GCs by mediating Dicer expression in PCOS ovaries. He et al. (2019) found that miRNA-200b/c are upregulated in GCs of PCOS patients, and then inhibit the proliferation of human granulosa cell KGN by targeting photosphatase and tense homolog (PTEN). Therefore, more and more evidence demonstrated that ncRNAs have an important regulatory function during the growth process of follicular GCs.

**FIGURE 4:**
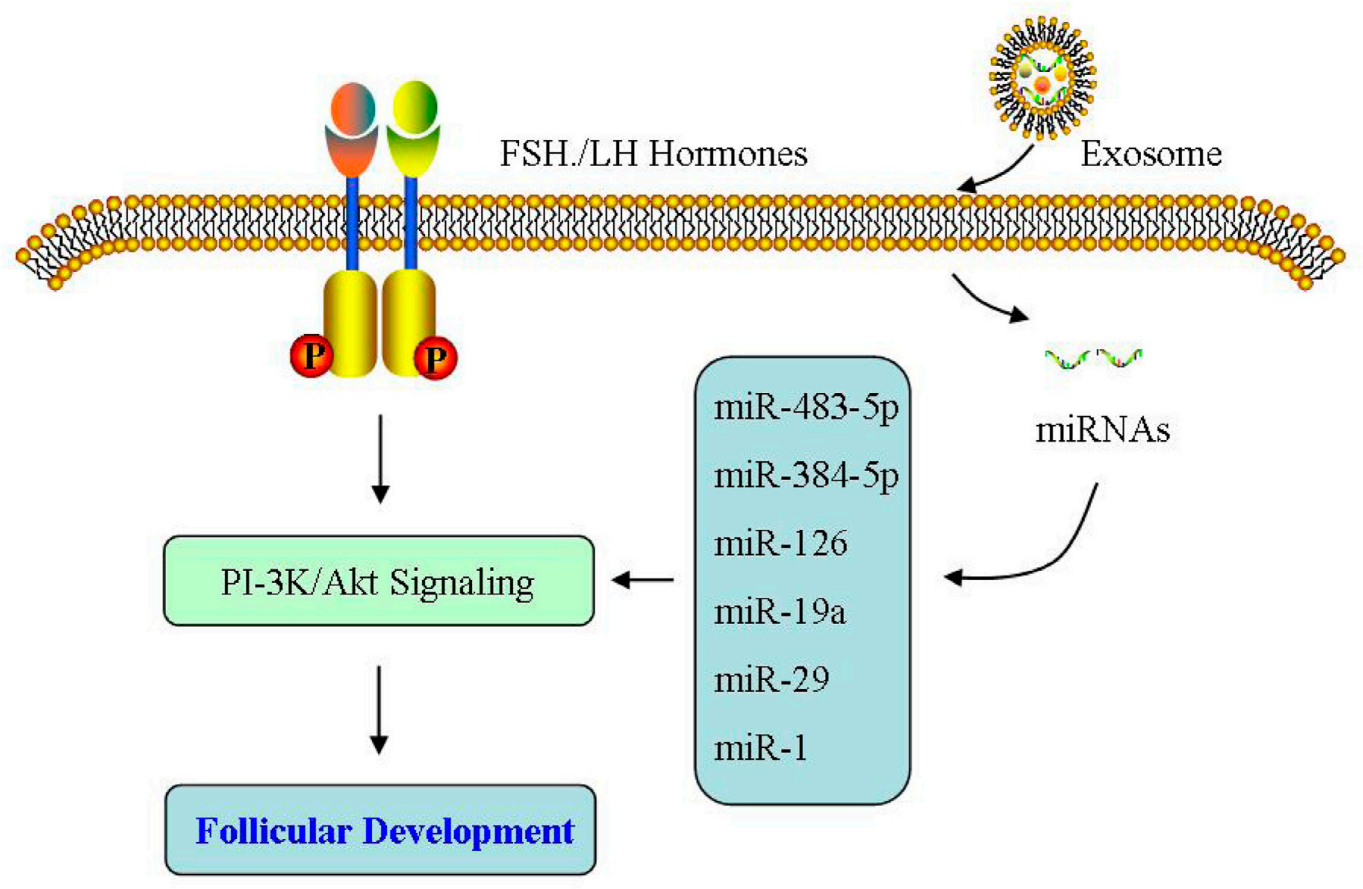
Effect of exosomes on follicular development. The exosomes derived from follicular fluid may participate in the development of PCOS as the novel molecules that regulate follicular development.

At the early stage of follicular development, oocytes exhibit an interdependent relationship with surrounding cumulus cells, where cumulus cells are mainly responsible for secreting growth hormone and ovarian steroid hormones, playing an important role in oocyte development. Cao et al. (2022a) reported that miR-143-3p/miR-155-5p secreted from follicular fluid can regulate follicular dysplasia through mediating the glycolysis of PCOS GCs. Zhao et al. (2019) showed that exosomal miR-323-3p is not only secreted from MSC, but also inhibits cumulus cell apoptosis by targeting PDCD4 in PCOS. In addition, Wang et al. (2019b) also confirmed that miR-323-3p is downregulated in cumulus cells of PCOS patients, and this downregulated miR-323-3p mediated steroid production by targeting IGF1. Therefore, exosomes derived from follicular fluid are highly likely to participate in the development of PCOS as novel molecules that regulate follicular development (Figure 4).

### 4.2 Effect of exosomes on hormone secretion

Hyperandrogenism is the main clinical symptom of PCOS, and recent studies have found a close correlation of miRNA and androgen production (Li et al., 2015; Fitzgerald et al., 2016; Cirillo et al., 2019; Luo et al., 2021). Murri et al. (2013) found through case control that miR-21, miR-27b, miR-103, and miR-155 in the blood of PCOS patients were significantly upregulated and positively correlated with the levels of free testosterone in the blood, affecting the release of testosterone. In addition, the levels of miR-222 and miR-146a in the blood of PCOS patients are correlated with serum insulin levels and testosterone concentrations (Murri et al., 2013). Rashad et al. (2019) reported serum miR-320 expressions were reduced in PCOS, especially with IR, and were negative with fasting insulin, PCOS phenotype, hirsutism score, ovarian volume, and sinus follicle number. Xu et al. (2015) detected significant differences in the expression of 59 miRNAs in PCOS GCs through miRNA expression profiling sequencing, and further bioinformatics analysis revealed that these differential expressed miRNAs can participate in important biological processes, such as regulating hormone metabolism and energy metabolism. Sang et al. (2013) detected the presence of multiple miRNAs in follicular fluid of PCOS patients, with significantly reduced expression levels of miR-132 and miR-320 related to estrogen metabolism. Yin et al. (2014) found that miR-383 in follicular fluid of PCOS patients was increased and also participated in steroid synthesis. Yao et al. (2018) conducted sequencing analysis on follicular fluid of PCOS patients and found 16 miRNA expressions were downregulated and 3 miRNA expressions were significantly upregulated. Among them, the abundance of miR-335-5p in PCOS was lower than that in non PCOS groups, and it was negative to the levels of anti-Mullerian, testosterone, and antral follicles. Given miR-200b as AR target, it is necessary for the ovulation regulated by HPO. miR-29c plays a role by influencing the downstream pathway of AR localization (Xue et al., 2018), indicating miR-200b/c involved in PCOS hyperandrogenism.

Cytochrome p450 enzymes, including CYP17, CYP21, CYP19, and CYP11A, are key enzymes in steroid hormone biosynthesis. Chaudhary et al. (2021) found that the increase in ovarian steroid production and androgen production is mainly due to the expression change of cytochrome p450 enzyme, a key enzyme in the steroid hormone biosynthesis pathway, in which *CYP11A* gene, as a possible genetic biomarker, functions in PCOS pathogenesis. Yu et al. (2021a) reported the levels of core enzyme mRNA involved in the steroid synthesis in PCOS exosomes. Among them, *CYP11A*, *CYP19A*, and *HSD17B2* mRNA levels increased. At the same time, with the change of hormone levels in follicular fluid, the levels of estriol, estradiol and isoprenol ketone in PCOS follicular fluid increased, and progesterone decreased, indicating that exosomal mRNA expression is related to the change of hormone levels in the follicular fluid (Yu et al., 2021a). The mRNA differential expressed in follicular fluid induces abnormal steroid production, which is the potential mechanism of the increase of estrogen and pregnenolone in PCOS follicular fluid (Yu et al., 2021a). However, due to the lack of comprehensive analysis of steroids in follicular fluid by researchers, further research is needed on the relationship between their expressions and androgen levels. Huang et al. (2020) revealed that the reduction of PCOS exosomal circLDLR can increase miR-1294 expression, inhibit *CYP19A1* expressions in receptor cells, and then decrease estrogen production. Interestingly, miRNA-199 was downregulated in PCOS exosomes, while it was high in PCOS follicular fluid. Considering that miRNA-199 is a potential target for CYP19A1, it is of great significance to detect and deeply explore the expression and role of exosomal miRNA-199 for studying the pathological mechanism of PCOS.

**FIGURE 5:**
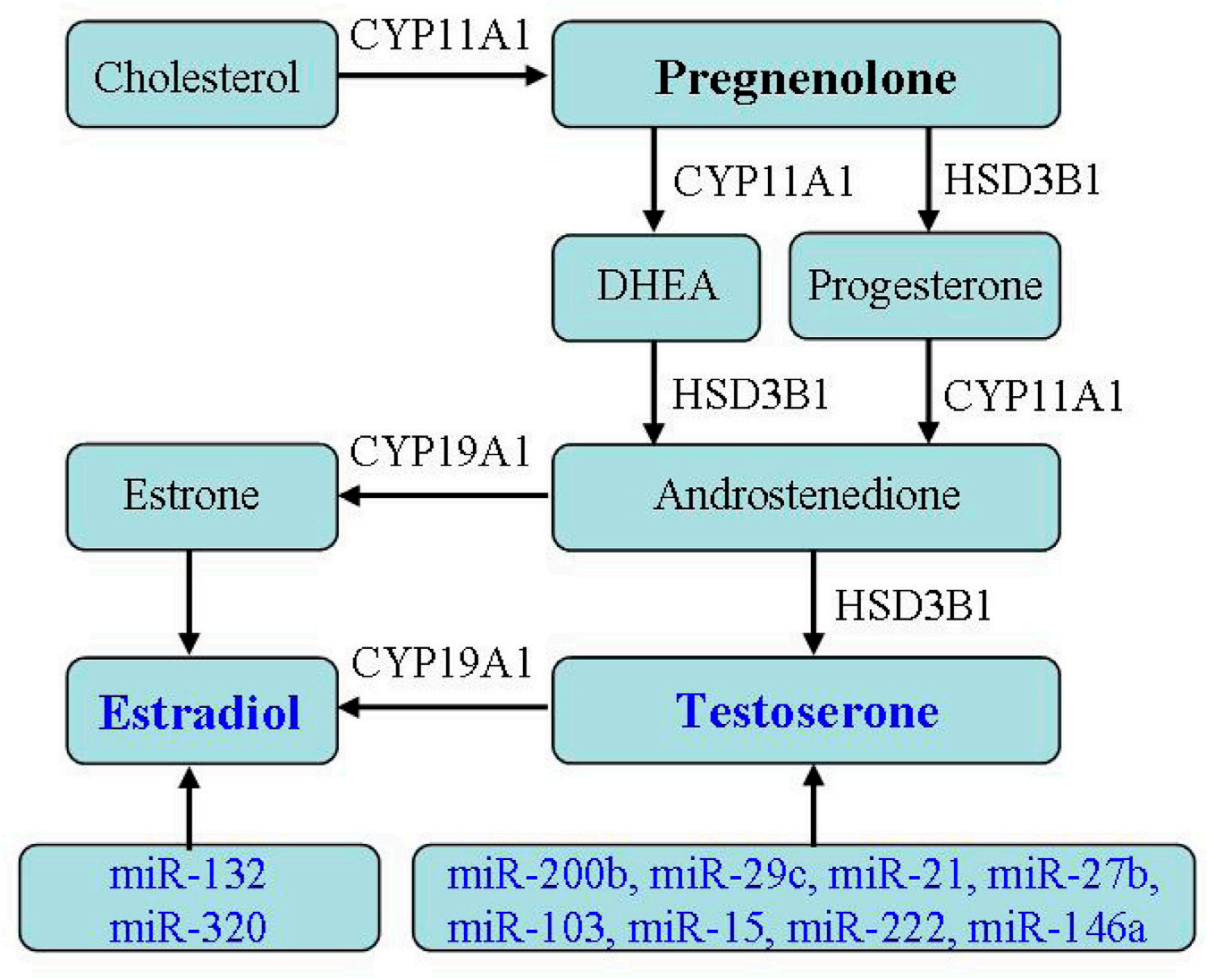
Effect of exosomes on hormone secretion. The relationship between PCOS exosomes and hormone secretion indicates exosomal miRNA regulating androgen secretion.

At present, there is not much research on the relationship between PCOS exosomes and hyperandrogenism. Further understanding miRNA regulating androgen secretion may help us improve PCOS clinical prognosis. However, there is currently a lack of in-depth research on how miRNA leads to PCOS androgen disorders (Figure 5).

### 4.3 Effect of exosomes on oxidative stress

PCOS is usually associated with obesity and impairs reproductive health, with a high sugar and fat diet being one of the causes of obesity. After administering a high fat and high sugar diet to letrozole-induced PCOS mice for a period of time, both the treatment and control group mice experienced severe metabolic disorders and reproductive disorders (Pieczynska et al., 2022). Excess accumulation of adipose tissue is the most prominent feature of obesity.Zhou et al. (2020b) reported that the exosomes derived from brown adipose tissue in young healthy mice can reduce the weight of obese mice induced by high-fat diet, reduce blood sugar and lipid accumulation and other metabolic syndromes under the exclusion of food intake. Hong et al. (2022) have shown that exosomal miR-20b-5p and miR-106a-5p can reduce adipocyte differentiation in a PCOS mouse model with IR, thereby alleviating lipid metabolism disorders in the pathogenesis of PCOS caused by IR. Katayama et al. (2019) reported that miR-20b-5p can regulate insulin-mediated glucose metabolism through PKB/Akt signaling to play an intracellular role. It is not only considered as a biomarker related to type 2 diabetes, but also a new molecule regulating glucose and lipid metabolism. From this, it can be seen that exosomes influence obesity by mediating the process of glucose and lipid metabolism, revealing the important role of exosomes in regulating lipid metabolism disorders in PCOS. It is widely known that one of the main factors of oxidative stress is ROS, which are active oxygen radicals that cause peroxidation and disorder in lipids and mutations in DNA, and exosomes play an important role in scavenger of these factors. Notably, the effect of exosomes on OS is still not fully understood in PCOS.

OS is closely related to lipid metabolism, and obesity and dyslipidemia, which are PCOS manifestations. Approximately 50% of PCOS women are overweight or obese (Glueck and Goldenberg, 2019). In PCOS women, miR-21, miR-27b, miR-103, and miR-155 were significantly higher in the obese than those in the control (Murri et al., 2013). Xiong et al. (2017) reported both of miR-23a/b reduced in PCOS, while miR-23b enhanced by raised BMI not miR-23a. Soh reported that miR-30c adjusts the levels of steroid and VLDL-C through decreased apolipoprotein, and becomes a therapeutic target for hyperlipidemia (Soh et al., 2013). At the same time, miR-122 can inhibit VLDL-C to mediate LDL-C contents (Rayner et al., 2010). The research also shows miR-122-5p/miR-223-3p is related to obese. On the contrary, miR-151a/miR-199a is negatively related to obese and WHR (Murri et al., 2018). miR-33 can decrease the expressions of ABCA1/ABCG1 and the contents of HDL, while blocking miR-33 can change VLDL-C and triglyceride contents through affecting cholesterol efflux and bile acid synthesis (Horie et al., 2010; Rayner et al., 2010; Aryal et al., 2017; Abdalla et al., 2020). Additionally, circulating LDL-C can also be affected by some specific exosomal miRNAs (Iacomino and Siani, 2017). miR-143 can decrease PKB/Akt activity mediated by insulin (Jordan et al., 2011). On the contrary, miR-375 promotes adipocyte differentiation via mediating the activity of PPARγ And ERK (Ling et al., 2011). All of these indicate the expression change of cell-specific miRNAs is intimately related to BMI and dyslipidemia, suggesting it as the metabolic targets in PCOS.

**FIGURE 6:**
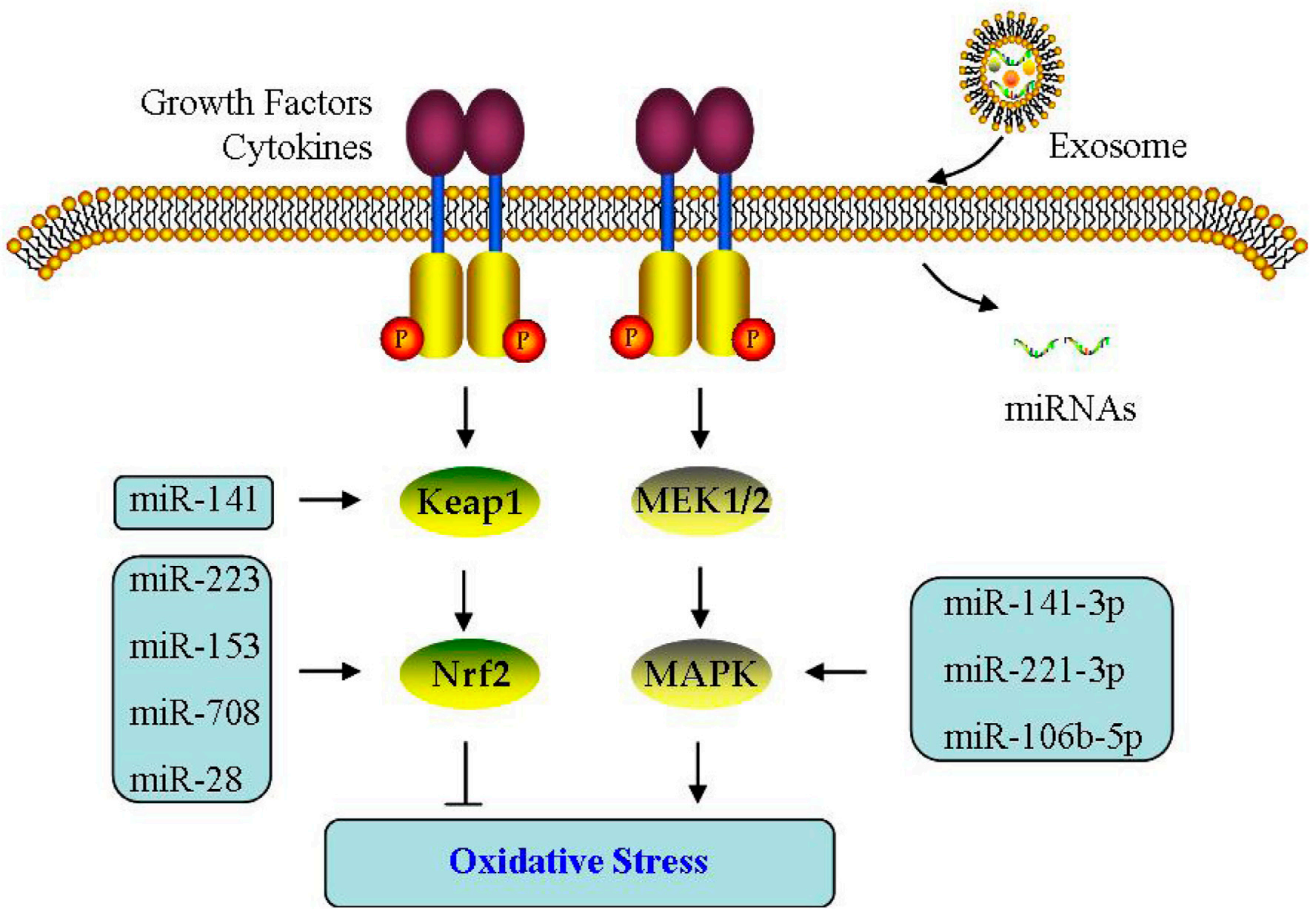
Effect of exosomes on oxidative stress. The effect of exosomal miRNA on oxidative stress is through Keap1/Nrf2 or MAPK signaling pathway in PCOS.

Interestingly, OS is undermined and apoptosis is decreased when Nrf2 signaling activated in PCOS (Ling et al., 2011). Simultaneously, miR-223 activates Nrf2 pathway through blocking Keap1 and increasing antioxidant systems (Ashrafizadeh et al., 2020). Notably, miR-141 inhibits Keap1 expression, stimulates Nrf2 signaling, reduces ROS level, and then improves OS (Zhou et al., 2019). Omar et al. found that in H_2_O_2_-treated bovine GCs, not only Nrf2 expression increased, but miR-28/miR-153/miR-708 expressions decreased; If these miRNAs are upregulated separately, it leads to the following decreased Nrf2 and antioxidant capacity, indicating the contribution of miRNAs to Nrf2-mediated OS (Khadrawy et al., 2019) (Figure 6).

### 4.4 Effect of exosomes on inflammatory response

Exosomes are considered as the key signal molecule carriers for the transmission of biomacromolecules during the inflammatory process, thus affecting the metabolism of target cells in many diseases, including PCOS (Zhang et al., 2019a; Console et al., 2019; Zhou et al., 2020a; Bouchareychas et al., 2020). For example, miR-155 is mediated for delivery by exosomes, which is involved in acute lung inflammation (Jiang et al., 2019). Exosomal lipids can restore membrane raft function and barrier integrity in IBD (Bowie et al., 2012). Exosomal proteins can affect brain inflammation (Yuan et al., 2017). Exosomal S100-A9 can also promote PCOS inflammation (Li et al., 2020a). In addition, IL-35 encapsulated in exosomes is a key molecule that inhibits inflammatory response (Kang et al., 2020). Recently, increasing researches demonstrate NLRP3 is also mediated through exosomes (Bai et al., 2019; Liang et al., 2020; Si et al., 2021). UMSC-Exo can reduce lysed-caspase 1 production, and then diminish IL-1β/IL-18 release and following pyroptosis (Yang et al., 2019). UMSC-Exo circHIPK3 decreases miR-421, leading to increased FOXO3a, expression, which can inhibit NLRP3 activation (Yan et al., 2020). These findings have already indicated the relationship between exosomes and inflammatory response, which maybe exist in PCOS similarly. Therefore, NLRP3 inflammasomes may also participate in PCOS pathogenesis.

The exosomes can monitor PD1 and TLR4/NF-κB/NLRP3 inflammasomes to regulate immunity in diseases (Yang et al., 2018; Dai et al., 2020). Dai et al. (2020) reported miR-148a can significantly inhibit TXNIP/TLR4 expression, leading to NF-κB/NLRP3 inflammasomes were suppressed, thereby alleviating myocardial IRI. Exosomal miR-126 inhibit NLRP3 activation and reduce hyperglycemia induced inflammatory response by reducing HMGB1 (Zhang et al., 2019b). Tang et al. (2020) found exosomal miR-320b can attenuate pyroptosis directly through suppressing NLRP3 inflammasomes during myocardial IRI. Exosomal miRNA-223 are inhibitors of NLRP3 inflammasome formation, which can inhibit NF-κB-mediated inflammatory response of macrophages (Haneklaus et al., 2013). Yu et al. (2021b) reported miR-21 can activate TLR8, leading to TNFα/IL-12 levels elevated in PCOS GCs. Knocking out miR-146a can increase pro-inflammatory gene expression and induce NF-κB activation (Runtsch et al., 2019).

**FIGURE 7:**
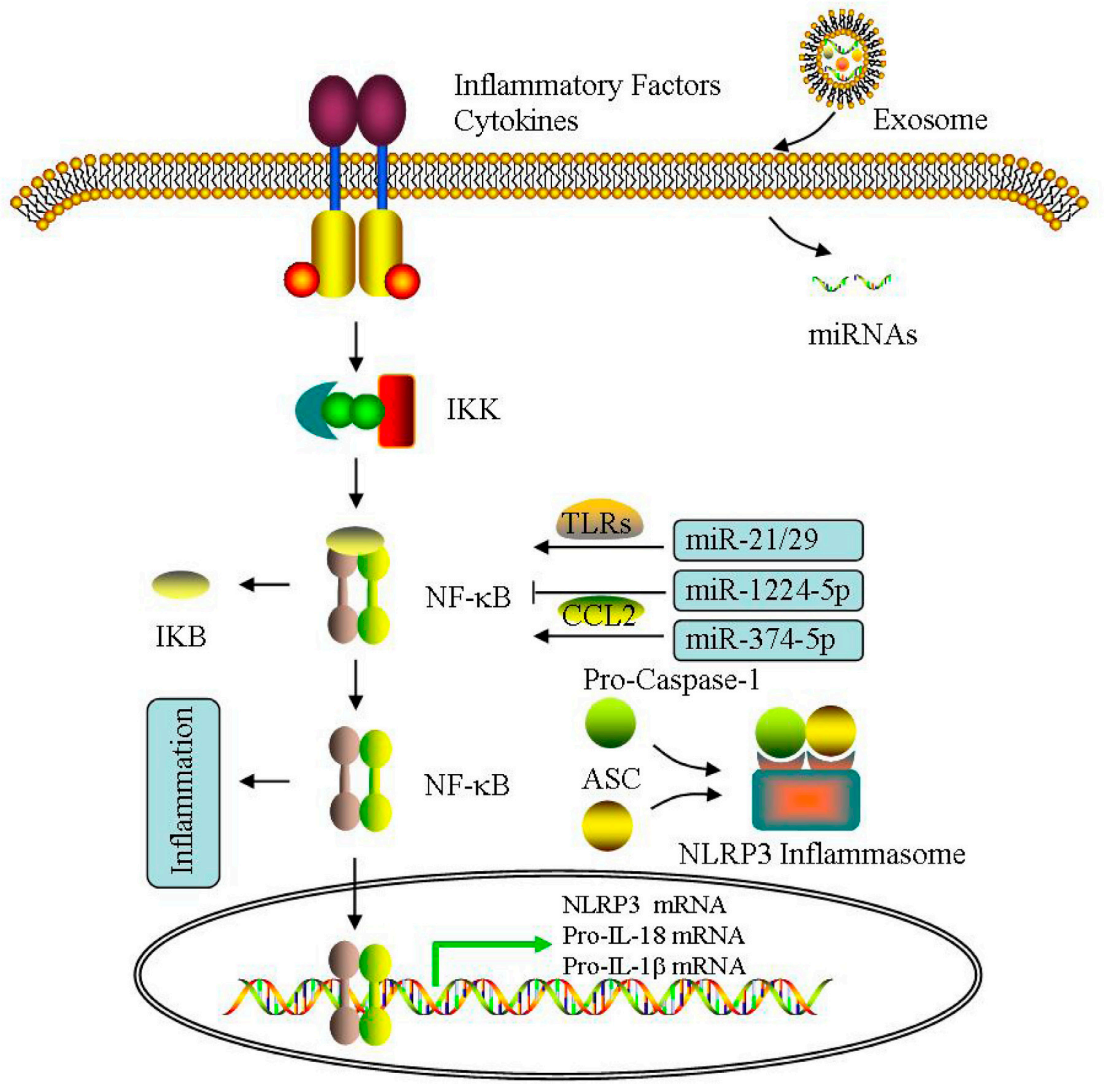
Effect of exosomes on inflammatory response. The effect of exosomal miRNA on inflammatory response is mainly through NF-κB pathway, including NLRP3 inflammasome activation in PCOS.

Additionally, NF-κB can activate CCL2, which is a main target of miR-374a-5p, indicating miR-374a-5p involved in inflammatory response (Doumatey et al., 2018). Li et al. (2021b) found miR-1224-5p can inhibit NF-κB signaling and then alleviates PCOS inflammation (Figure 7).

### 4.5 Effect of exosomes on insulin resistance

Insulin resistance is commonly present in PCOS and also involved in its reproductive and metabolic complications (Diamanti-Kandarakis and Dunaif, 2012; Bannigida et al., 2020). Sun et al. (2018c) have shown that exosomes derived from human MSCs can reverse peripheral IR and alleviate *β* cell destruction to alleviate type 2 diabetes. The ectopic lipid accumulation in the liver has received attention as one of the possible factors affecting PCOS metabolic syndrome (Hwang et al., 2022). Gao et al. (2022) studied the liver tissue and exosomes of mice at different stages of PCOS, showing the complex metabolism between liver tissue and exosomes, in which the downregulation of glycolysis and tricarboxylic acid cycle may be related to the liver pathophysiological mechanism of PCOS.

In the PCOS rat model induced by dehydroepiandrosterone, AMSC and its derived exosomes are regulated via miR-21-5p, which can control glucose homeostasis by targeting BTG2, and significantly reduce multiple phenotypes in PCOS (Cao et al., 2022b). Ye et al. (2022) conducted transcriptome and metabonomics conjoint analysis on exosomes of PCOS patients, and found that β-tocopherol, 1-methyl hydantoin and 2-isopropyl malic acid may be involved in PCOS pathogenesis. The miRNA sequencing results showed differential miRNAs can control the contents of differential metabolites, which may serve as potential biomarkers for diagnosing PCOS. Therefore, it can be seen that exosomes can regulate the metabolic process of peripheral tissues including liver and adipose tissue between organs through the expression of regulator gene, thus affecting IR, and then mediating PCOS.

**FIGURE 8:**
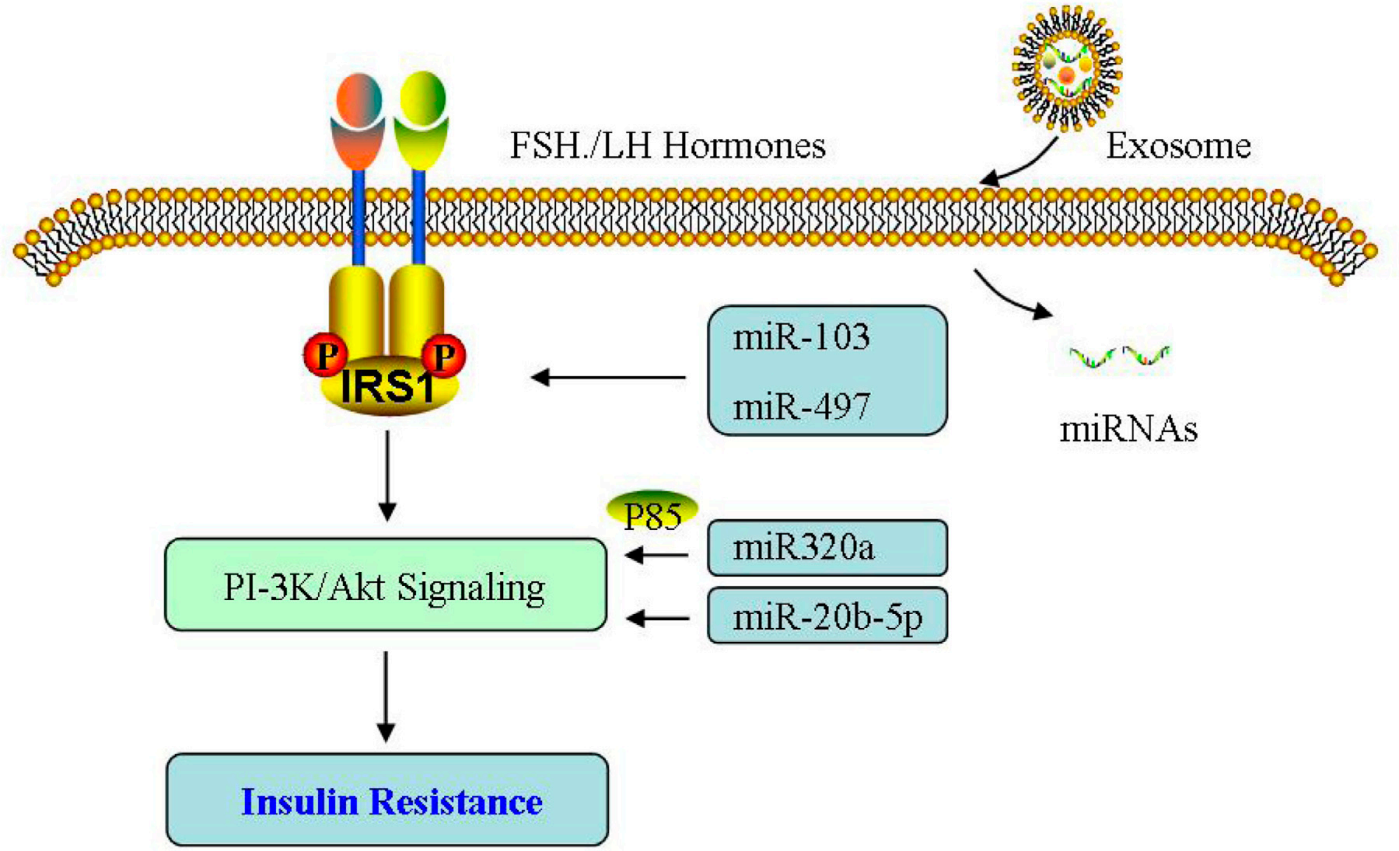
Effect of exosomes on insulin resistance. The effect of exosomal miRNA on insulin resistance is mainly through PI-3K/Akt signaling pathway in PCOS.

In addition, the contribution of miRNAs to IR has been widely investigated in PCOS (Sorensen et al., 2014; Cirillo et al., 2019; Bahmyari et al., 2021; Yu et al., 2021b), demonstrating that glucose metabolism is abnormal and miR-194/miR-193b/miR-122 are obviously increased. In PCOS only with IR, miR-223 expression is enhanced, which can induce GLUT4 expression. miR-1333a and miR-133b are involved in GLUT4 expression via KLF15, and attenuate glucose utilization for IR control. Given the regulatory relationship between miRNA and GLUT4, GULT4 may also be another novel target during PCOS therapy (Chakraborty et al., 2014; Gebremedhn et al., 2021). Furthermore, there are a negative relationship of miR-146a with IR, TNFα and IL-6, while Jiang et al. (2021) reported exosomal miR-146a has no association with glucose metabolism indicators (Balasubramanyam et al., 2011). miR-24 is reduced without a relationship with abnormal IR and hormones in PCOS (Nanda et al., 2020). Overall, miRNA may function as another potential novel target during PCOS therapy (Figure 8).

## 5 Therapeutic potential of exosomes in PCOS

In recent years, the research of exosomes as a new drug carrier applied to targeted therapy of diseases has attracted wide attention, among which exosomes based disease diagnosis and drug research and development have made rapid progress in tumor, cardio cerebral vascular disease, diabetes, neurodegenerative diseases and other major diseases (Haney et al., 2015; Jansen et al., 2017; Console et al., 2019; Peng et al., 2020; Kimiz-Gebologlu and Oncel, 2022).Katakowski et al. (2013) transfected miR-146b plasmid into MSCs to obtain exosomes rich in miR-146b, and then injected them into polymorphous keratinoblastoma in mice. The results showed that the treatment method of using exosomes to transport miRNA can inhibit the growth of mouse tumor cells. Another study found that the exosomes of MSCs rich in miR-133b increased the level of outward growth of neurite *in vitro*, and eliminated the downregulation of miR-133b in the brain induced by stroke, indicating that the treatment of stroke with exosomes containing specific miRNA can promote brain remodeling (Johnsen et al., 2014; Chen and Chopp, 2018; Li et al., 2021a). In addition, exosomes derived from MSCs can selectively deliver a specific miRNA let-7c to damaged kidneys, thereby up-regulating the expression level of let-7c and reducing renal fibrosis (Lu et al., 2018). Based on the advantages and functional characteristics of exosomes in drug delivery, exosomes and their differential expressed contents have played an advantageous role in treating follicular development, metabolic abnormalities, and alleviating inflammation in PCOS.

Phosphatidylinositol 3-kinase (PI-3K) signaling pathway functions in PCOS through influencing ovarian follicular development and hormone secretion (Wu et al., 2015; Wu et al., 2016; Wang et al., 2017a; Wang et al., 2017b; Tang et al., 2017; Lu et al., 2018; Zhang et al., 2020a; Tang et al., 2021). For example, Zhou et al. (2022) proved miR-18b-5p produced by exosomes derived from follicular fluid can reduce PTEN expression and promote PI-3K/Akt/mTOR signaling activation, thereby improving PCOS. MAPK/Nrf2 signaling pathway participates in energy metabolism and antioxidant defense system pf ovarian cells of PCOS (Khadrawy et al., 2019; Zhou et al., 2019; Ashrafizadeh et al., 2020; Jiang et al., 2021). For example, ROS activates the MAPK pathway and can be blocked by antioxidants, while H_2_O_2_ induces an increase in ROS, inhibits specific miRNAs and activates MAPK pathway (Zhao et al., 2022b). NF-κB can mediate pro-inflammatory factor expression, which is critical for the following inflammatory response. For example, Zhao et al. (2022b) reported that exosomes derived from human umbilical cord MSCs can inhibit NF-κB signaling in PCOS, thereby reducing ovarian GC inflammatory response and increasing anti-inflammatory factor IL-10 expression, while also inhibiting the pro-inflammatory factor TNFα and interferon-γ expression, decreasing cell apoptosis, and promoting progesterone production.

**FIGURE 9:**
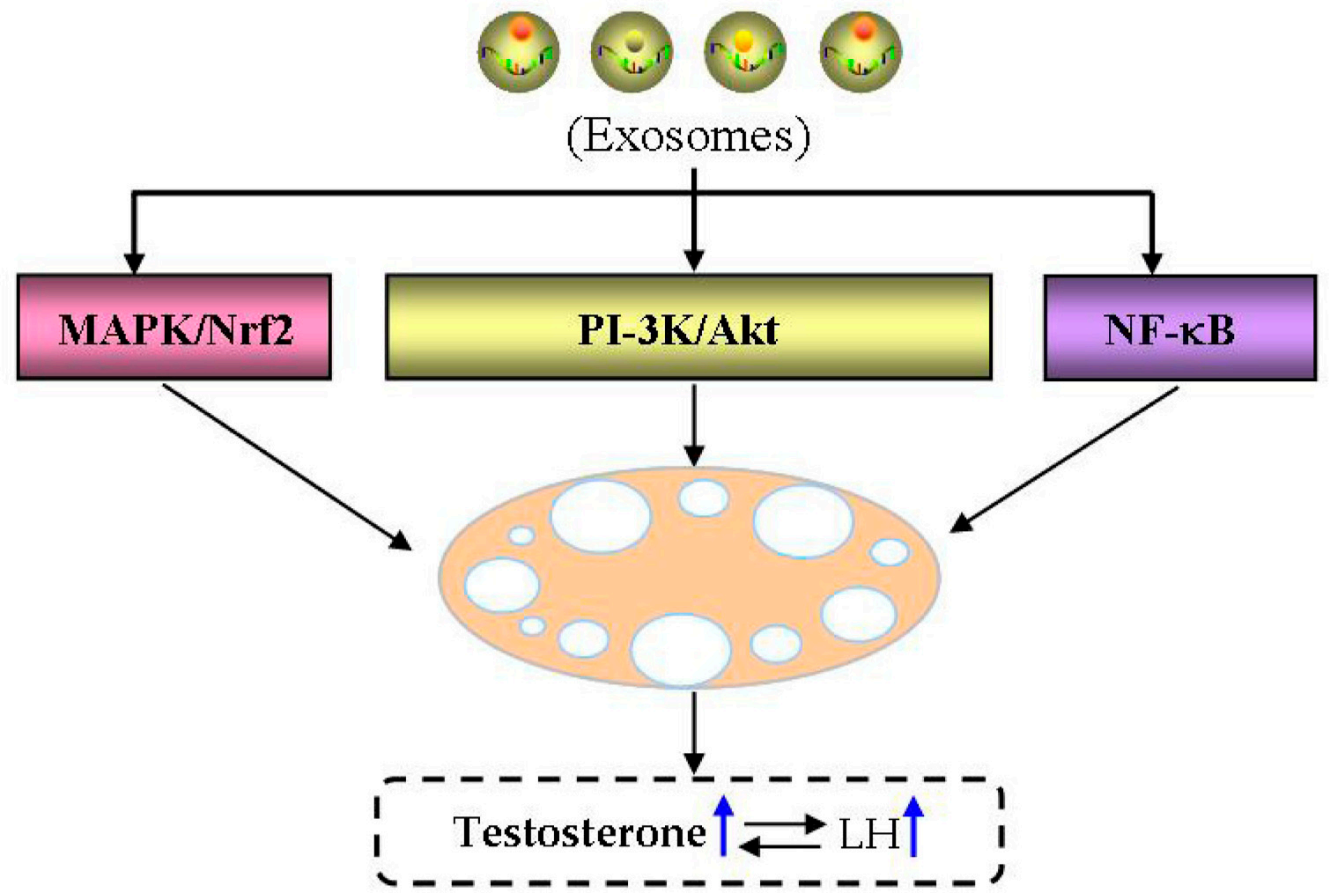
Therapeutic potential of exosomes in PCOS. The therapeutic potential of exosomes in PCOS is main through MAPK/Nrf, PI-3K/Akt and NF-kB signaling pathways.

Notably, the conditioned medium is considered as a putative and rich source of exosomes in clinical settings, and the importance of cell vs. cell-free therapy has been investigated in the field of reproductive biology both in human and animal settings (Jiao et al., 2020; Zhang et al., 2021b; Abd-Elwahab et al., 2023; Amiri et al., 2023). For example, recent findings discussed the regenerative potential of exosomes in premature ovarian condition and even azoospermia in animal settings (Li et al., 2021c; Nazdikbin Yamchi et al., 2023; Park et al., 2023). Therefore, exosomes and their non coding RNAs may serve as diagnostic and therapeutic targets for PCOS, especially MSC-derived exosomes as a new method for treating infertility caused by PCOS (Figure 9).

## 6 Summary and prospects

In conclusion, investigations about exosomes have developed rapidly, involved various fields about physiological projects. Exosomes have been successfully used as a circulating biomarker for breast cancer, ovarian cancer and other cancers, and as a tumor-related molecule before metastasis (Pant et al., 2012; Peinado et al., 2017; Sun et al., 2018b; Mashouri et al., 2019; Pegtel and Gould, 2019). At the same time, it also serves as a potential marker for some diseases, such as central nervous system degeneration and Alzheimer’s disease (Fais et al., 2016; Kanninen et al., 2016), and is currently attempting to convert exosomes into promising vaccines for treating diseases (Lasser, 2015). Exosomes play a crucial role in many biosynthetic processes, and the RNA and protein content they carried can reflect the level of their originating cells. Therefore, quantitative research on exosomes is crucial in future research (Sun et al., 2018a), especially for early diagnosis and treatment of diseases.

The special endocrine state in PCOS is often close to the onset of spontaneous abortion, pregnancy diabetes, pregnancy hypertension and other diseases (Palomba et al., 2015; Wang et al., 2016; Anagnostis et al., 2018; Azziz, 2018). Due to the significant heterogeneity of PCOS, treatment plans typically provide personalized guidance, exercise guidance, and ovulation promoting drugs, but pregnancy outcomes are often unsatisfactory. As a multifunctional bio-active carrier, exosomes are involved in cell signaling transduction. Due to their stable nature, compared with general biomarker, exosomes can provide a large number of specificity and sensitivity indicators (Di Pietro, 2016). Therefore, by analyzing the changes in protein or nucleic acid profiles of exosomes and selecting molecules specifically expressed as biomarkers for the diagnosis and prognosis of PCOS, which may become a novel approach for early PCOS diagnosis.

In addition, differential miRNA expression in the blood, ovaries and exosomes has special significance for the diagnosis, treatment, and prognosis of PCOS. Exosomes with multiple molecules serve as important carriers of inter-cellular communication, participating in PCOS by mediating follicular development, hormone content, insulin resistance, and metabolic disorders, in order to study the therapeutic effect of exosomes and their contents according to the source of cells, and analyze the related factors and pathways of exosomes acting on PCOS, which is helpful to further explore the pathogenesis of PCOS. With the help of bioengineering technology, exosomes are expected to be used as carriers to deliver chemicals, RNA, and proteins. In the future, targeted therapy of PCOS may be realized, so as to alleviate the endocrine and metabolic abnormalities of PCOS patients, to achieve the goal of treating infertility caused by PCOS.

Finally, research on exosomes is still at the early stages, and large-scale production and storage of exosomes are difficult. Currently, there are no standardized methods for exosomal separation and analysis. However, it is believed that with the deepening of researches on exosomal bioengineering and the exploration of their biochemical and physicochemical properties through various emerging technologies, the large-scale application of exosomes in medical practice will be further developed. Furthermore, it provides novel clues for PCOS research and applications.

## Figures and Tables

**Table:** 

**ABCA1**	ATP binding cassette transporter A1
**ABCG1**	ATP binding cassette subfamily G member 1
**AID**	autoimmune disease
**AMSCs**	adipose derived mesenchymal stem cells
**AR**	androgen receptor
**BMI**	body mass index
**BMPRA**	bone morphogenetic protein receptor 1 A
**BTG2**	B cell translocation gene 2
**CDCA4**	cell division cycle associated 4
**CDKN1A**	cyclin dependent kinase inhibitor 1 A
**circRNA**	circular RNA
**CVD**	cardiovascular disease
**CYP19A1**	cytochrome p450 family 19 subfamily A member 1
**ERK**	extracellular signal-regulated kinase
**ERS**	endoplasmic reticulum stress
**EV**	extracellular vesicle
**FOXO3a**	forkhead box class O3a
**GCs**	granulosa cells
**GLUT4**	glucose transporter 4
**HDL**	high-density lipoprotein
**HMGB1**	high mobility group box-1
**HPA**	hypothalamic-pituitary-adrenal axis
**HPO**	hypothalamic-pituitary-ovarian axis
**IBD**	inflammatory bowel disease
**IGF1**	insulin-like growth factor 1
**IL-35**	interleukin-35
**IR**	insulin resistance
**IR**	insulin resistance
**IRI**	ischemia/reperfusion injury
**KLF15**	kruppel like transcription factor 15
**LDL-C**	low-density lipoprotein cholesterol
**MAPK**	mitogen activated protein kinase
**miRNA**	microRNA
**MSCs**	mesenchymal stem cells
**mTOR**	mammalian target of rapamycin
**NAFLD**	non-alcoholic fatty liver disease
**ncRNA**	non coding RNA
**OS**	oxidative stress
**PCOS**	polycystic ovary syndrome
**PD1**	programmed cell death 1
**PDCD4**	programmed cell death 4
**PKB/Akt**	protein kinase B
**S100-A9**	S100 calcium-binding protein A9
**SHBG**	sex hormone-binding globulin
**siRNA**	small interfering RNA
**TLR8**	toll like receptor 8
**UMSC-Exo**	umbilical cord mesenchymal stem cell derived exosomes
**VLDL-C**	very low-density lipoprotein cholesterol
**WHR**	waist-hip ratio
